# A Two Dimensional Overlapped Subaperture Polar Format Algorithm Based on Stepped-chirp Signal

**DOI:** 10.3390/s8053438

**Published:** 2008-05-26

**Authors:** Xinhua Mao, Daiyin Zhu, Xin Nie, Zhaoda Zhu

**Affiliations:** Department of Electronic Engineering, Nanjing University of Aeronautics and Astronautics, Nanjing, 210016, China

**Keywords:** Synthetic aperture radar (SAR), subaperture, synthetic bandwidth, stepped-chirp signal, space-variant phase compensation

## Abstract

In this work, a 2-D subaperture polar format algorithm (PFA) based on stepped-chirp signal is proposed. Instead of traditional pulse synthesis preprocessing, the presented method integrates the pulse synthesis process into the range subaperture processing. Meanwhile, due to the multi-resolution property of subaperture processing, this algorithm is able to compensate the space-variant phase error caused by the radar motion during the period of a pulse cluster. Point target simulation has validated the presented algorithm.

## 1. Introduction

Synthetic aperture radar (SAR) becomes an important tool in modern remote sensing for its all-weather, day and night capability to provide high-resolution maps of scene of interest. The demand for radar images is constantly pushing for finer resolutions. This quest for the resolving power has two major consequences [1-2]: first, their useful bandwidth should increase in proportion with the resolution in range. Second, the length of the synthetic antenna should increase in proportion with the along-track resolution.

Due to technical limitations, particularly the limited sampling rate of the analog to digital converters, synthetic bandwidth technique [3-6] is proposed to solve the hardware challenges of wideband radar. As compared to the commonly used wideband linear frequency modulated (LFM) radar waveform, by adopting stepped-chirp waveform and applying synthetic bandwidth techniques, it is possible to achieve high range resolution while still retaining the advantages of lower instantaneous receiver bandwidth and lower analog-to-digital sampling rate. However, the relative motion between the radar and the target during the period of a pulse cluster is not always negligible. If it is not taken into account, as a consequence, it may result in performance degradations, such as range error, loss in signal-to-noise ratio, and degraded range resolution. Unfortunately, the phase errors resulted from this motion are space-variant. The traditional synthetic bandwidth techniques only compensate these errors corresponding to a specified point target, for example, the scene center. Consequently, the purpose of this paper is to compensate this space-variant phase error.

With respect to the azimuth dimension, high resolution is obtained by coherent integration over a large aperture. The generally linear radar flight trajectory assumption, which is the basis of frequency domain image formation algorithm, is deviated, especially when nonplanar motion (NPM) occurs. Polar format algorithm (PFA) [7-8] is a popular high resolution spotlight SAR algorithm due to its efficient NPM compensation capability. However, due to the planar wavefront approximations made in PFA, the data exhibits space-variant phase errors and migration errors, which limit the focused scene size. Then, subaperture algorithm [9] is proposed. Due to its space-variant phase correction capability, fine resolution imaging of large scene becomes available.

In this paper, a new image formation algorithm which incorporates the synthetic bandwidth technique with subaperture processing is proposed. Instead of traditional pulse synthesis preprocessing, it integrates the pulse synthesis into range subaperture processing. Meanwhile, it is able to compensate the space-variant phase errors caused by the radar motion during the pulse cluster.

## 2. SAR Signal Modeling

**Figure 1.**
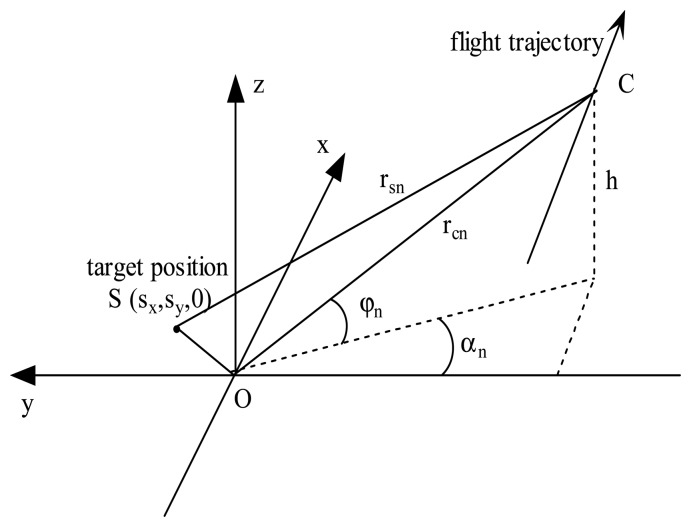
SAR geometry.

Consider a spotlight SAR operating in the geometry of Figure 1, for simplicity, we only examine the case in which the sensor travels in a straight line at constant velocity. The central point of the scene is defined as the origin of the coordinate system, and radar antenna phase center (APC) is identified by coordinates (*x*, *y*, *z*). The variable *α_n_* and *φ_n_* are the APC's instantaneous squint angle and grazing angle, respectively, at the nth LFM pulse. They equal *α*_0_ and *φ*_0_ at the center of the aperture. The distance from the APC to the scene center is *r_cn_*. In this paper, we account for the broadside mode only, then *α*_0_ = 0. A target scatter is located at (*s_x_*, *s_y_*, 0), and the range from APC to this target is measured by *r_sn_*. After performing dechirp and residual video phase (RVP) elimination on the echo signal of the nth pulse, we can express the Doppler phase history as [7]

(1) $$f\left(\;n,i\right)=\exp\left\{j\frac{4\pi }{c}\left(f_{0}+\gamma T_{s}i\right)R_{\Delta }\right\}$$

where *R*_Δ_ = (*r_cn_*−*r_sn_*) is the differential range, *c* is the velocity of light, *f*_0_ is the carrier frequency of the transmitted signal, *γ* is the chirp rate, *T_s_* is the sample interval in fast time, *i* is the range sample index with −*I*/2≤*i*≤ *I/*2 − 1, and *n* is the index value of pulse number with −*N*/2 ≤ *n* ≤ *N*/2 −1.

The differential range *R*_Δ_ can be expressed as [7]

(2) $$\begin{array}{ll} R_{\Delta } & =s_{x}\cos\varphi_{n}\sin\alpha_{n}-s_{y}\cos\varphi_{n}\cos\alpha_{n}+r_{e}\\ & =\cos\varphi_{n}\cos\alpha_{n}\left(s_{x}\tan\alpha_{n}-s_{y}+\xi \left(n\right)\right) \end{array}$$

where $\xi \left(n\right)=\frac{r_{e}}{\cos\varphi_{n}\cos\alpha_{n}}$, which is caused by the assumption of planar wavefront. By using the Taylor expansion, *ξ*(*n*) can be expressed as *ξ* (*n*) ≈ *ε*_0_ + *ε*_1_*n* + *ε*_2_*n*^2^ when the cubic and higher order terms are ignored. Since the coefficients of this polynomial are dependent on the target position, this error is space-variant.

Inserting Equation (2) into Equation (1), the sampled signal can be represented as

(3) $$\begin{array}{ll} f\left(n,i\right) & =\exp\left\{j\frac{4\pi }{c}\left(f_{0}+\gamma T_{s}i\right)\cos\varphi_{n}\cos\alpha_{n}\left(s_{x}\tan\alpha_{n}-s_{y}+\xi \left(n\right)\right)\right\}\\ & =\exp\left\{j\kappa_{n}\left(1+\beta_{0}i\right)\;\left(s_{x}\tan\alpha_{n}-s_{y}+\xi \left(n\right)\right)\right\} \end{array}$$

where $\kappa_{n}=\frac{4\pi f_{0}}{c}\cos\varphi_{n}\cos\alpha_{n}$ and $\beta_{0}=\frac{\gamma T_{s}}{f_{0}}$.

Performing range resampling formulated by

(4) $$\kappa_{n}\left(1+\beta_{0}i\right)=\kappa_{0}\left(1+\beta_{0}i\right)$$

where $\kappa_{0}=\frac{4\pi f_{0}}{c}\cos\varphi_{0}\cos\alpha_{0}$ is a constant, we can have the phase history as

(5) $$f_{r}\left(n,í\right)=\exp\left\{j\kappa_{0}\left(1+\beta_{0}í\right)\left(s_{x}\tan\alpha_{n}-s_{y}+\xi \left(n\right)\right)\right\}.$$

For the purpose of clearness and simplicity, we still use *i* instead of *í* in the following discussion. Then, Equation (5) is expressed as

(6) $$f_{r}\left(n,i\right)=\exp\left\{j\kappa_{0}\left(1+\beta_{0}i\right)\left(s_{x}\tan\alpha_{n}-s_{y}+\xi \left(n\right)\right)\right\}$$

If the space sampling position *n* satisfied tan *α_n_* = *dαn*, where *dα* is constant, the range resampled signal can be modeled as

(7) $$f_{r}\left(n,i\right)=\exp\left\{j\kappa_{0}\left(1+\beta_{0}i\right)\left(s_{x}d\alpha n-s_{y}+\xi \left(n\right)\right)\right\}.$$

## 3. Two Dimensional Overlapped Subaperture Polar format Algorithm (PFOSA) [9]

Equation (7) is the phase history after range resampling. In full aperture PFA image formation, we get the image by performing an azimuth resampling followed by a 2-D DFT, or an azimuth chirp-z transform (CZT) followed by a range DFT. However, due to the space-variant phase error term *ξ*(*n*), the focused scene size of interest is constrained to be very small in ultra-high resolution SAR. Subaperture algorithm, which can provide coarse resolution images before the final fine resolution image formation, has been proposed to overcome this constraint [9]. Due to the coarse information of the individual scatter's location extracted from the coarse resolution images, the compensation of space-variant phase error becomes applicable. In the following, we briefly review of the PFOSA proposed in [9].

First, we divide the azimuth and range aperture into subapertures, respectively, by making

(8) $$\begin{array}{l} n=m_{1}+\Delta_{2}m_{2}\\ i=k_{1}+\mu_{2}k_{2} \end{array}$$

where *m*_1_ is the azimuth intra-subaperture index limited within −*M*_1_ /2≤ *m*_1_ ≤ *M*_1_ /2 − 1, *m*_2_ is the azimuth inter-subaperture index limited within −*M*_2_/2≤*m*_2_≤*M*_2_/2 − 1, Δ_2_ is the azimuth data decimation factor, *k*_1_ is the range intra-subaperture index limited within −*K*_1_ /2 ≤ *k*_1_ ≤ *K*_1_ / 2 − 1, *k*_2_ is the range inter-subaperture index limited within −*K*_2_/2≤*k*_2_ ≤*K*_2_/2−1, and *m*_2_ is the range data decimation factor. Using Equation (8), we rewrite the Equation (7) as

(9) $$f_{r}\left(m_{1},m_{2};k_{1},k_{2}\right)=\exp\left\{j\kappa_{0}\left(1+\beta_{0}\left(k_{1}+\mu_{2}k_{2}\right)\right)\left(s_{x}d\alpha \left(m_{1}+\Delta_{2}m_{2}\right)-s_{y}+\xi \left(m_{1}+\Delta_{2}m_{2}\right)\right)\right\}.$$

Next, applying the quadratic order approximation of *ξ*(*n*) and rearrange Equation (9) following the index *m*_1_, *k*_1_, *m*_2_, *k*_2_ sequentially, we get

(10) $$\begin{array}{l} f_{r}\left(m_{1},m_{2};k_{1},k_{2}\right)=\exp\left\{j\kappa_{0}\left(-s_{y}+{ɛ}_{0}\right)\right\}\\ \cdot \exp\left\{j\kappa_{0}\left(1+\beta_{0}\left(k_{1}+\mu_{2}k_{2}\right)\right)\left(\left(s_{x}d\alpha +{ɛ}_{1}+2{ɛ}_{2}\Delta_{2}m_{2}\right)m_{1}+{ɛ}_{2}m_{1}^{2}\right)\right\}\\ \cdot \exp\left\{j\kappa_{0}\beta_{0}\left(s_{x}d\alpha \Delta_{2}m_{2}-s_{y}+{ɛ}_{0}+{ɛ}_{1}\Delta_{2}m_{2}+{ɛ}_{2}{\left(\Delta_{2}m_{2}\right)}^{2}\right)k_{1}\right\}\\ \cdot \exp\left\{j\kappa_{0}\left(1+\beta_{0}\mu_{2}k_{2}\right)\left(\left(s_{x}d\alpha \Delta_{2}+{ɛ}_{1}\Delta_{2}\right)m_{2}+{ɛ}_{2}{\left(\Delta_{2}m_{2}\right)}^{2}\right)\right\}\\ \cdot \exp\left\{j\kappa_{0}\beta_{0}\mu_{2}\left(-s_{y}+{ɛ}_{0}\right)k_{2}\right\} \end{array}$$

where, the first term is a constant, which is neglected in the following discussion. The second and third terms are the azimuth and range intra-subaperture terms, which correspond to coarse resolution image. The fourth and fifth terms are the inter-subaperture terms, which correspond to fine resolution image. Also note that each exponential term contains some undesired error phase. In the subaperture terms, we choose subaperture size *M*_1_ and *K*_1_ in such a way that the phase error terms caused by wavefront curvature in the subaperture can be neglected. It is helpful to note that error terms in the last two terms can be compensated due to space position information extracted from the coarse resolution images. Now to facilitate the analysis, we rewrite Equation (10) as following

(11) $$\begin{array}{ll} f_{r}\left(m_{1},m_{2};k_{1},k_{2}\right) & =\exp\left\{j\kappa_{0}\left(1+\beta_{0}\left(k_{1}+\mu_{2}k_{2}\right)\right)\left(s_{x}d\alpha m_{1}\right)\right\}\\ & \cdot \exp\left\{j\kappa_{0}\beta_{0}\left(-s_{y}\right)k_{1}+j\phi_{e1}\right\}\\ & \cdot \exp\left\{j\kappa_{0}\left(1+\beta_{0}\mu_{2}k_{2}\right)\left(s_{x}d\alpha \Delta_{2}\right)m_{2}+j\phi_{e2}\right\}\\ & \cdot \exp\left\{j\kappa_{0}\beta_{0}\mu_{2}\left(-s_{y}\right)k_{2}+j\phi_{e3}\right\} \end{array}$$

where *ϕ_e_*_1_ = *κ*_0_*β*_0_*s_x_dα*Δ_2_*m*_2_*k*_1_, *ϕ_e_*_2_ = *κ*_0_ (1 + *β*_0_*μ*_2_*k*_2_)(*ε*_1_Δ_2_*m*_2_+*ε*_2_ (Δ_2_*m*_2_)^2^), and *ϕ_e_*_3_ = *κ*_0_*β*_0_*m*_2_*ε*_0_*k*_2_ are the undesired terms, which should be compensated in this algorithm.

From Equation (11), the processing strategy is clear. First, perform a CZT across *m*_1_, following by phase correction *ϕ_e_*_1_, and then perform a DFT across *k*_1_ to get the coarse resolution images. After the second phase error compensation *ϕ_e_*_2_, we perform a CZT across *m*_2_ to get azimuth fine resolution. Finally, after compensating the third phase error term *ϕ_e_*_3_, a DFT across *k*_2_ results in the final fine resolution image.

## 4. Stepped-Chirp based PFOSA (SCPFOSA)

To reduce the transmission bandwidth, and meanwhile to achieve the high range resolution, it is possible to transmit series of narrow-band signals centered at different carrier frequencies. For example, an equivalent wideband LFM chirp can be assembled from lesser-bandwidth chirp segments in the data processing stage. These subchirp signals, which are referred to as a pulse cluster, are transmitted as separate pulses, each with their own carrier frequencies. The carrier frequencies distribute sequentially to keep the spectrums covering the desired bandwidth.

Now assume that each pulse cluster has *K*_2_ chirp segments each with bandwidth *B_s_*, the carrier frequency of the middle subchirp is *f*_0_, and the step carrier frequency is Δ*f* (Δ*f* < *B_s_*). Then the center frequency of the *k*_2_ th (−*K*_2_/2≤*k*_2_ ≤ *K*_2_/2−1) subchirp is *f*_0_ + *k*_2_Δ*f*. Analogous to Equation (3), after preprocessing, the *k*_2_th backscattered echo signal in the *n*th pulse cluster can be represented by

(12) $$f\left(n,k_{1},k_{2}\right)=\exp\left\{j\frac{4\pi }{c}\left(f_{0}+k_{2}\Delta f+\gamma T_{s}k_{1}\right)\cos\varphi_{n,k_{2}}\cos\alpha_{n,k_{2}}\left(s_{x}\tan\alpha_{n,k_{2}}-s_{y}+\xi \left(n,k_{2}\right)\right)\right\}$$

where *k*_1_ is the range sample index in each chirp segment, *k*_2_ is the chirp segment index, and *n* is the cluster index. It is important to note that the grazing angle *φ_n,k_*_2_ and squint angle *α_n,k_*_2_ vary not only with index *n*, which is desirable, but also with *k*_2_, which is undesirable. The latter change of *φ_n,k_*_2_ and *α_n,k_*_2_ is resulted from the radar motion during the pulse cluster. Neglecting this variation will introduce space-variant phase errors which limit the focused scene size. But in Equation (12) it does not appear explicitly. To illustrate this effect, we develop a polynomial expression by using Taylor series expansion.

The key to analysis of the characteristics of the Equation (12) is to have expression for cos*φ_n,k_*_2_ cos*α_n,k_*_2_ in terms of tan *α_n,k_*_2_. From the geometry in Figure.1, it is easy to get the following relationship

(13) $$\cos\varphi_{n,k_{2}}\cos\alpha_{n,k_{2}}=\frac{1}{\sqrt{1+\tan^{2}\varphi_{0,0}+\tan^{2}\alpha_{n,k}}}$$

where *φ*_0,0_ is the grazing angle at the aperture center corresponding to *α_n,k_*_2_ = 0.

Since the wavefront curvature error term *ξ*(*n*, *k*_2_) does not play an important role in this development, it is neglected. Then inserting Equation (13) into Equation (12) and perform a Taylor series expansion, we get the signal phase history in Equation (12) approximated as

(14) $$\Phi \;□\kappa_{o}[1+\beta_{0}\left(k_{1}+\mu_{2}k_{2}\right)]\;\left(s_{x}\tan\alpha_{n,k_{2}}-s_{y}+\frac{1}{2}s_{y}\cos^{2}\varphi_{0,0}\tan^{2}\alpha_{n,k_{2}}\right)$$

where $\kappa_{o}=\frac{4\pi f_{0}}{c}\cos\varphi_{0,0}$, $\beta_{0}=\frac{\gamma T_{s}}{f_{0}}$ and $\mu_{2}=\frac{\Delta f}{\gamma T_{s}}$.

As before, we assume that the space sampling position *n* satisfies tan*α_n,k_*_2_ = *dα*(*nK*_2_+*k*_2_) then Equation (14) can be divided into two parts

(15) $$\Phi =\Phi_{\text{basic}}+\Phi_{\text{err}}$$

where

(16) $$\begin{array}{l} \Phi_{\text{basic}}=\kappa_{o}[1+\beta_{0}\left(k_{1}+\mu_{2}k_{2}\right)]\;\left[s_{x}d\alpha nK_{2}-s_{y}+\frac{1}{2}s_{y}\cos^{2}\varphi_{0,0}{\left(d\alpha \right)}^{2}{\left(nK_{2}\right)}^{2}\right]\\ \Phi_{\text{err}}=\kappa_{o}[1+\beta_{0}\left(k_{1}+\mu_{2}k_{2}\right)]\;\left[s_{x}d\alpha k_{2}+\frac{1}{2}s_{y}\cos^{2}\varphi_{0,0}{\left(d\alpha \right)}^{2}\left(2nK_{2}k_{2}+k_{2}^{2}\right)\right] \end{array}$$

Phase term Φ*_basic_* contains the basic imaging information. The phase term Φ*_err_*, which causes distortion and defocus in range, is the error term introduced by the radar motion during pulse cluster. Due to its dependence on target position (*s_x_*, *s_y_*), this phase error is space-variant. Analogous to phase error resulted from wavefront curvature, if not compensated, it set the focused scene size limit, and particularly troublesome as resolution approaches the nominal wavelength of the radar.

For Equation (12), after range resampling, the phase history becomes

(17) $$f_{r}\left(n,k_{1},k_{2}\right)=\exp\left\{j\kappa_{o}[1+\beta_{0}\left(k_{1}+\mu_{2}k_{2}\right)]\;\left[s_{x}\tan\alpha_{n,k_{2}}-s_{y}+\xi \left(n,k_{2}\right)\right]\right\}$$

where $\kappa_{o}=\frac{4\pi f_{0}}{c}\cos\varphi_{0,0}$, $\beta_{0}=\frac{\gamma T_{s}}{f_{0}}$ and $\mu_{2}=\frac{\Delta f}{\gamma T_{s}}$.

Comparing with Equation (6), it is clear to see that in Equation (17) we get the range subaperture data naturally via the transmission and reception of the chirp segment signals. The difference is that *α_n,k_*_2_ and *ξ*(*n*, *k*_2_) varies with index *k*_2_, while in Equation (6) they keep constant at specified *n*.

Insert tan*α_n,k_*_2_ = *dα*(*nK*_2_ + *k*_2_) into Equation (17), and then divide the azimuth aperture into subapertures

(18) $$\begin{array}{c} f_{r}\left(m_{1},m_{2};k_{1},k_{2}\right)=\exp\left\{j\kappa_{0}\left[1+\beta_{0}\left(k_{1}+\mu_{2}k_{2}\right)\right]\;\left[s_{x}d\alpha \left(K_{2}\left(m_{1}+\Delta_{2}m_{2}\right)+k_{2}\right)-s_{y}+\xi \left(m_{1},m_{2},k_{2}\right)\right]\right\}\\ =\exp\left\{j\kappa_{0}\left[1+\beta_{0}\left(k_{1}+\mu_{2}k_{2}\right)\right]\;\left[s_{x}d\alpha K_{2}\left(m_{1}+\Delta_{2}m_{2}\right)-s_{y}+\xi \left(m_{1},m_{2},k_{2}\right)\right]\right\}\cdot \exp\left\{j\phi_{err}\left(k_{1},k_{2}\right)\right\} \end{array}$$

where *ϕ_err_*(*k*_1_, *k*_2_) = *κ_o_* [1 + *β*_0_(*k*_1_ + *μ*_2_*k*_2_)] *s_x_dαk*_2_ is the phase error term resulted from radar motion during pulse cluster but after range resampling. Compared with phase error term Φ*_err_* before range resampling, parts of phase error are compensated, and the range position dependence is eliminated.

**Figure 2.**
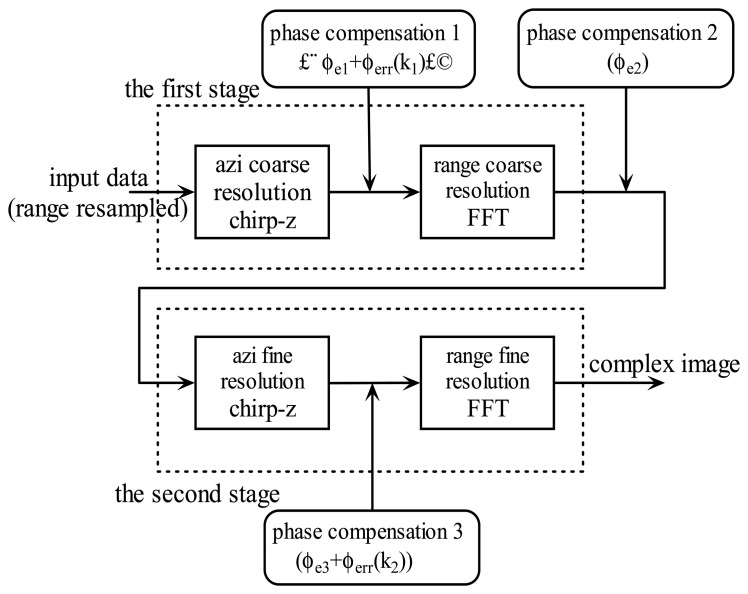
Flow chart of SCPFOSA.

Analogous to Equation (11), for Equation (18), we neglect the constant phase term and wavefront error terms in the subapertures and rearrange the rest terms

(19) $$\begin{array}{ll} f_{r}\left(m_{1},m_{2};k_{1},k_{2}\right) & =\exp\left\{j\kappa_{0}\left[1+\beta_{0}\left(k_{1}+\mu_{2}k_{2}\right)\right]\left(s_{x}d\alpha K_{2}m_{1}\right)\right\}\\ & \cdot \exp\left\{j\kappa_{0}\beta_{0}\left(-s_{y}\right)k_{1}+j\phi_{e1}+j\phi_{err}\left(k_{1}\right)\right\}\\ & \cdot \exp\left\{j\kappa_{0}\left(1+\beta_{0}\mu_{2}k_{2}\right)\left(s_{x}d\alpha K_{2}\Delta_{2}\right)m_{2}+j\phi_{e2}\right\}\\ & \cdot \exp\left\{j\kappa_{0}\beta_{0}\mu_{2}\left(-s_{y}\right)k_{2}+j\phi_{e3}+j\phi_{\text{err}}\left(k_{2}\right)\right\} \end{array}$$

where *ϕ_e_*_1_ = *κ*_0_*β*_0_*s_x_dαK*_2_Δ_2_*m*_2_*k*_1_, *ϕ_e_*_2_ = *κ*_0_ (1+*β*_0_*μ*_2_*k*_2_) [*ε*_1_ Δ_2_*m*_2_ + *ε_2_* (Δ_2_*m*_2_)^2^], and *ϕ_e_*_3_ = *κ*_0_*β*_0_*μ*_2_*ε*_0_*k*_2_ are undesired terms just like those in PFOSA which are introduced by wavefront curvature. While error phase terms *ϕ_err_*(*k*_1_) = *κ_o_β*_0_*s_x_dαk*_2_*k*_1_ and $\phi_{err}\left(k_{2}\right)=\kappa_{o}s_{x}d\alpha k_{2}+\kappa_{o}\beta_{0}\mu_{2}s_{x}d\alpha k_{2}^{2}$, the two parts of *ϕ_err_*(*k*_1_,*k*_2_), are due to radar motion during the pulse cluster. These error terms are space-variant due to the dependence on azimuth position (range dependence is eliminated owing to range resampling). If these phase errors are not compensated, as a consequence, they result in displacement and defocus in range. Since the coarse location information can be extracted from the coarse resolution images, it is possible to compensate these errors by modifying the classical PFOSA. The new algorithm (we call it SCPFOSA) can be derived from Equation (19), whose flow chart is illustrated in figure 2.

Step1: Perform a CZT across *m*_1_, get the azimuth coarse resolution estimate *s_x_*.
Step2: Use the estimate of *s_x_* to compensate the error phase terms *ϕ_e_*_1_ and *ϕ_err_* (*k*_1_), and then perform a FFT across *k*_1_ to obtain the range coarse resolution estimate *s_y_*.
Step3: Use the estimate of *s_x_* and *s_y_* to correct the error term *ϕ_e_*_2_, then perform a CZT across *m*_2_ to get the azimuth fine resolution estimate *s_x_*.
Step4: Use the fine resolution estimation of *s_x_* and coarse resolution estimate of *s_y_* to compensate the error terms *ϕ_e_*_3_ and *ϕ_err_* (*k*_2_), and then perform a FFT across *k*_2_ to get the range fine resolution estimate *s_y_*. The result is the fine resolution complex SAR image.

## 5. Simulation Results

**Figure 3.**
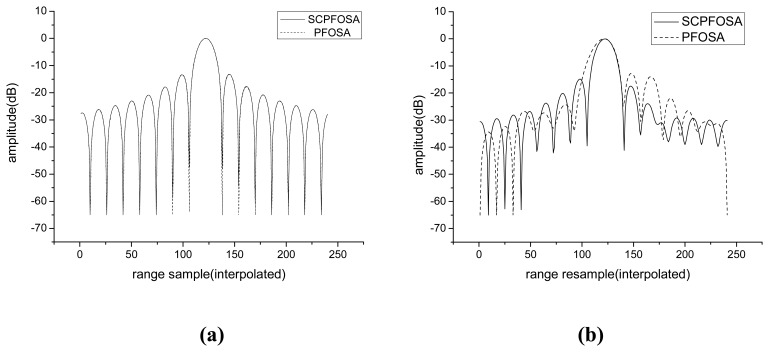
Comparison of SCPFOSA and PFOSA. **(a)** Range profile of the IRF for scene center point. **(b)** Range profile of the IRF for azimuth displaced target.

In this section, point target simulation is employed to validate the presented algorithm. The waveform parameters are chosen as: *B_s_* = 250*MHz*, Δ*f* =100*MHz*, *K*_2_ =15. The other parameters are listed as follow: standoff range is 10km, azimuth resolution is 0.1m, and radar forward velocity is 150m/s. Two point targets are simulated, the first one is the scene center point, and the other one is located at azimuth 150m away from the scene center. The new algorithm is evaluated with respect to the classical PFOSA which doesn't compensate the error terms resulted from the use of stepped-chirp signals. In subaperture algorithm, subapertures are overlapped to control the sidelobes; in particular, they are overlapped to control the amplitude to grating lobes due to data decimation. The degree of allowable overlap will depend on the window functions employed, and sidelobe toleration limits. In our paper, the overlap rate is not the problem we are concerned, so we do not employ window function in the simulation. As the phase error term *ϕ_err_* (*k*_1_, *k*_2_) results in distortion and defocus only in the range, we show the range profiles of impulse response function (IRF) for the two simulated targets (Figure. 3). For scene center point, since the error terms *ϕ_err_* (*k*_1_) and *ϕ_err_* (*k*_2_) are both zeros, the two algorithms have almost the same response. However, for the azimuth displaced point target, the mainlobe of range profile is broadening for PFOSA, since the phase errors caused by motion of radar during pulse cluster are not compensated. While using SCPFOSA, due to the correction of these phase errors, its range profile has improved significantly (mainlobe reduce 12% and peak sidelobe ratio (PSR) reduces about 2.5dB).

## 6. Conclusion

In this paper, a 2-D subaperture algorithm based on stepped-chirp signal is presented. It integrates the pulse synthesis process into range subaperture processing without traditional pulse synthesis preprocessing. Meanwhile, due to the multi-resolution property of subaperture processing, this algorithm is able to compensate the space-variant phase error resulted from the motion of radar during a pulse cluster. SCPFOSA has almost the same processing flow chart with PFOSA, only the additional phase error term are added, it has the comparable computation complexity with PFOSA. Furthermore, due to its repetitive architecture in subaperture processing, the SCPFOSA is very suitable for parallel and pipeline hardware architectures.
